# Adjunctive Low-Level Laser Therapy and Deep Oscillation in Pulmonary Rehabilitation for Post-Tuberculosis Lung Disease

**DOI:** 10.3390/life16101682

**Published:** 2026-10-08

**Authors:** Marin Vlăduț Piciorea, Gabriela-Marina Andrei, Mihai Olteanu, Daniela Matei, Magdalena Rodica Trăistaru

**Affiliations:** 1 Doctoral School, University of Medicine and Pharmacy of Craiova, 200349 Craiova, Romania; marin.piciorea@umfcv.ro; 2 Internal Medicine—Department of Pulmonology, University of Medicine and Pharmacy of Craiova, 200349 Craiova, Romania; gabriela.andrei@umfcv.ro (G.-M.A.); mihai.olteanu@umfcv.ro (M.O.); 3 Department of Physiotherapy, Faculty of Nursing, University of Medicine and Pharmacy of Craiova, 200349 Craiova, Romania; rodica.traistaru@umfcv.ro

**Keywords:** post-tuberculosis lung disease, low-level laser therapy, deep oscillation, pulmonary rehabilitation

## Abstract

Background: Post-tuberculosis lung disease (PTLD) is associated with persistent functional impairment and reduced quality of life. This study evaluated the association between the addition of low-level laser therapy (LLLT) and deep oscillation (DO) to structured pulmonary rehabilitation and changes in health-related quality-of-life and functional outcomes in patients with clinically stable PTLD. Methods: In this prospective, controlled, non-randomized study, 65 patients completed an eight-week program. The study group (SG; *n* = 33) received structured kinesiotherapy plus LLLT and DO, while the control group (CG; *n*= 32) received the same kinesiotherapy alone. Between-group effects were assessed using baseline-adjusted ANCOVA with HC3 robust standard errors and Benjamini–Hochberg correction. Results: Greater improvements were observed in the combined-intervention group across all prespecified outcomes (all *q* < 0.001). Adjusted between-group differences were 6.46 points (95% CI 5.73–7.19) for EUROHIS-QOL-8, 8.86 (7.39–10.33) for WHOQOL-BREF physical health, 14.69 m (10.57–18.81) for 6MWD, −0.62 s (−0.94 to −0.30) for TUG, −1.04 (−1.29 to −0.79) for mMRC, and −2.21 (−2.63 to −1.79) for Borg CR10. Conclusions: Adjunctive LLLT and DO were associated with greater improvements in quality-of-life and functional outcomes than kinesiotherapy alone. Given the non-randomized design and the combined administration of the two adjunctive modalities, these findings should be interpreted as associative and require confirmation in randomized, sham-controlled trials.

## 1. Introduction

Tuberculosis (TB) remains one of the leading infectious causes of morbidity and mortality worldwide despite major advances in microbiological diagnosis and antimicrobial treatment [1]. Although successful anti-tuberculosis therapy achieves microbiological cure, it frequently fails to restore normal respiratory function. Tuberculosis may precede, accompany, or result in a broad spectrum of chronic respiratory abnormalities collectively referred to as tuberculosis-associated lung diseases (TBALDs) [2]. Among these, post-tuberculosis lung disease (PTLD) has emerged as an important clinical entity and was formally defined by international Delphi consensus as persistent respiratory abnormality, with or without symptoms, attributable at least in part to previous tuberculosis after completion of anti-tuberculosis treatment [3,4]. Epidemiological evidence indicates that approximately 60% of TB survivors continue to experience persistent respiratory impairment, and many never recover their pre-disease pulmonary function despite bacteriological cure [5,6].

PTLD is a heterogeneous condition characterized by pulmonary fibrosis, fibro-cicatricial changes, bronchiectasis, residual cavitary lesions, small-airway disease, and ventilation–perfusion abnormalities, resulting in obstructive, restrictive, or mixed ventilatory defects [2,7]. Beyond structural lung damage, PTLD is associated with exercise intolerance, dyspnea, impaired mobility, reduced health-related quality of life, and increased long-term mortality [8,9].

Persistent inflammatory activity, abnormal tissue remodeling, altered respiratory mechanics, respiratory muscle dysfunction, skeletal muscle deconditioning, and physical inactivity collectively contribute to the maintenance of disability long after completion of antimicrobial treatment [2,10,11,12].

Because current anti-tuberculosis therapy eradicates Mycobacterium tuberculosis but does not reverse the structural and functional consequences of PTLD, pulmonary rehabilitation has become an increasingly important component of long-term management. Comprehensive rehabilitation combines exercise training, respiratory physiotherapy, patient education, and behavioural interventions to improve exercise capacity, symptom burden, functional independence, and health-related quality of life [13,14,15]. Functional outcomes are commonly evaluated using standardized measures of exercise capacity, mobility, dyspnea, perceived exertion, and health-related quality of life [14,15,16,17,18,19,20,21,22,23,24,25,26,27,28,29], whereas exploratory biomarkers such as NLR (neutrophil-to-lymphocyte ratio) and PLR (platelet-to-lymphocyte ratio) may provide additional information regarding persistent systemic inflammatory activity [20,21].

Although pulmonary rehabilitation is increasingly recommended for patients with PTLD, the available evidence remains considerably more limited than that for chronic obstructive pulmonary disease. Most published studies are single-centre investigations with relatively small sample sizes and few have evaluated adjunctive rehabilitation modalities beyond exercise training itself [22,23,24,25]. Nevertheless, recent evidence has shown that structured pulmonary rehabilitation improves pulmonary function, exercise capacity, oxygen saturation, and quality of life in patients with PTLD, further supporting its role as an essential component of post-tuberculosis care [25]. This observation is consistent with the broader rehabilitation literature, where structured non-pharmacological interventions have been shown to improve functional status and health-related quality of life across a range of chronic conditions, reinforcing the central role of active rehabilitation in long-term patient management [26].

Given the multifactorial mechanisms underlying disability in PTLD, adjunctive therapies capable of improving respiratory mechanics, reducing persistent inflammatory activity, optimizing soft-tissue function, and facilitating exercise performance may further enhance the benefits of conventional pulmonary rehabilitation. Low-level laser therapy (LLLT), also referred to as photobiomodulation (PBM) therapy, has demonstrated biological effects related to modulation of inflammatory pathways, mitochondrial activation, cellular energy metabolism, oxidative stress reduction, and tissue repair [27,28]. In chronic respiratory disorders, limited evidence suggests that photobiomodulation applied to the thoracic region or respiratory muscles may improve dyspnea and functional exercise capacity, although consistent improvements in pulmonary function or respiratory muscle performance have not been demonstrated [29,30,31]. Deep oscillation (DO), an electrostatic low-frequency oscillation therapy, has been associated with improved microcirculation, lymphatic drainage, soft-tissue mobility, and tissue elasticity, but its potential contribution to pulmonary rehabilitation has received little scientific attention and has not been specifically investigated in patients with PTLD [32,33,34,35].

Rather than reversing irreversible pulmonary lesions, the complementary physiological effects of PBM therapy and DO may improve chest wall mobility, respiratory mechanics, soft-tissue flexibility, symptom control, and tolerance to physical exercise, thereby creating more favorable conditions for exercise-based rehabilitation [29,30,31,32,33,34,35]. This multimodal approach may enhance the functional benefits achieved through structured kinesiotherapy while remaining fully consistent with the current understanding of PTLD pathophysiology.

We hypothesized that the addition of combined LLLT and DO to an identical structured kinesiotherapy program would be associated with greater improvements in health-related quality-of-life and functional outcomes than structured kinesiotherapy alone in patients with PTLD. This hypothesis was based on the potential complementary role of these adjunctive modalities in facilitating symptom control, soft-tissue mobility, and tolerance to active exercise, rather than on an expected reversal of the underlying structural pulmonary abnormalities.

The primary objective of this study was to evaluate the association between the addition of combined LLLT and DO to structured kinesiotherapy and changes in health-related quality of life in patients with clinically stable PTLD. Health-related quality of life was assessed using two complementary measures: the EUROHIS-QOL-8, which provides a brief multidimensional assessment of overall quality of life, and the Physical Health domain of the WHOQOL-BREF, which more specifically captures physical functioning, energy and fatigue, mobility, pain and discomfort, sleep and rest, dependence on medical treatment, and work capacity. The use of these complementary measures was intended to capture both broader perceived health-related quality of life and the physical-health dimension most directly relevant to a rehabilitation intervention. Secondary objectives were to evaluate associations with changes in functional exercise capacity, functional mobility, dyspnea, and perceived exertion. Changes in NLR and PLR were additionally examined as exploratory systemic inflammatory indices.

To our knowledge, no published clinical study has specifically evaluated PBM therapy applied to the chest wall or the combined use of LLLT and DO as adjuncts to structured kinesiotherapy in patients with PTLD. Consequently, the present study should be considered an exploratory clinical evaluation of a combined adjunctive rehabilitation strategy. Given its non-randomized design and the concurrent administration of LLLT and DO, the study was not designed to establish the independent efficacy of either modality or their individual causal contributions to the observed outcomes.

## 2. Materials and Methods

### 2.1. Study Design

This prospective, controlled, non-randomized interventional study was conducted between January and June 2026 at the Department of Pneumology of the “Victor Babeș” Clinical Hospital of Infectious Diseases and Pneumophthisiology and the Department of Physical Medicine and Rehabilitation of Filantropia Hospital, Craiova, Romania. The study protocol was approved by the Ethics Committee of the University of Medicine and Pharmacy of Craiova (Authorization no. 9/10 January 2025), and all participants gave their written informed consent to participate in the study.

No formal a priori sample-size calculation was performed; the initial cohort of 76 participants represented all consecutive eligible patients who could be recruited during the predefined study period. Therefore, the recruitment number reflected the available clinical population rather than a statistically determined target sample size. Seventy-six consecutive patients diagnosed with PTLD were recruited for the study, based on the inclusion criteria. Participants were allocated to the two groups using a non-random procedure based on their willingness and ability to attend the supervised rehabilitation sessions and on the availability of the adjunctive treatment procedures. Consequently, group allocation was not concealed and may have introduced selection bias. Random allocation was not feasible in this clinical setting because access to the adjunctive LLLT and DO procedures depended on the availability of the treatment modalities and on each participant’s ability and willingness to attend the supervised rehabilitation sessions. Therefore, assignment could not be performed independently of these practical constraints and was not based on a randomly generated allocation sequence. Forty patients were recruited for the study group (SG), which received the institutionalized scheme of kinesiotherapy combined with LLLT and DO, and thirty-six patients were recruited for the control group (CG), which received the same scheme of kinesiotherapy, but without the additional electrotherapy procedures.

An eight-week individualized, supervised inpatient and outpatient post-TB pulmonary rehabilitation program was administered to the participants of both groups. Participants continued their previously prescribed pharmacological treatments for PTLD and comorbidities throughout the study period. Pharmacological management was individualized according to clinical indications; therefore, individual medication regimens were not necessarily identical across participants. However, the same therapeutic classes used for the management of PTLD were represented in both study groups, and no changes to these treatments were introduced as part of the study protocol during the eight-week follow-up.

**Figure 1:**
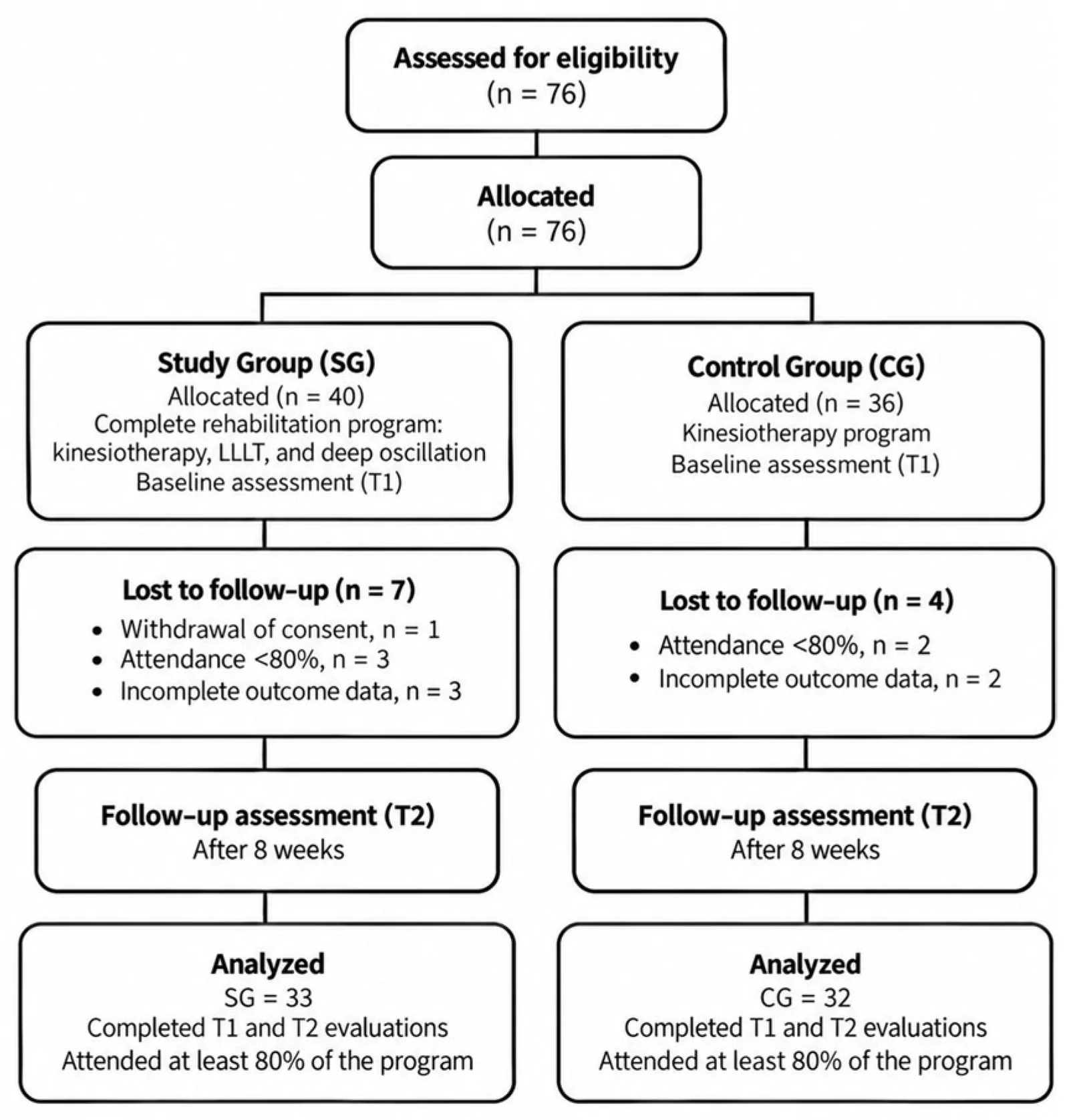
Diagram of study.

Clinical, functional, laboratory, and quality-of-life assessments were performed at the baseline (T1)—before the intervention and at the end of the eight-week rehabilitation intervention (T2). The selection process and flow of participants are presented in Figure 1 below.

Accordingly, the study was designed and analyzed as a prospective, controlled, non-randomized interventional study, and the observed between-group differences were interpreted as associations rather than as evidence of causality. To address this limitation, in all statistical analyses of intergroup comparisons, the possible confounding effect of baseline imbalances was adjusted for using the methods described in Section 2.5.

### 2.2. Study Population

The final analytic sample comprised 65 patients with PTLD who had completed anti-tuberculosis treatment and had persistent respiratory impairment. For statistical accuracy, only participants who completed both evaluations, attended at least 80% of the rehabilitation program, and provided valid data were included in the final analysis. In the SG, 33 patients (82.5%) completed the prescribed program and attended the follow-up evaluation, while in the CG, 32 patients (88%) completed the follow-up evaluation (Figure 1). Of the seven participants from the study group who were not included in the final analysis, one withdrew consent, three attended fewer than 80% of the prescribed rehabilitation sessions, and three had incomplete outcome data. Of the four participants from the control group who were not included in the final analysis, two attended fewer than 80% of the prescribed sessions and two had incomplete outcome data.

#### 2.2.1. Inclusion and Exclusion Criteria

Inclusion criteria were: age ≥18 years; documented history of pulmonary tuberculosis with completed anti-tuberculosis treatment and no evidence of active disease at enrollment; persistent respiratory impairment consistent with PTLD, including obstructive, restrictive, or mixed ventilatory dysfunction; clinical stability for at least four weeks before enrollment; ability to participate safely in an exercise-based pulmonary rehabilitation program; ability to understand the study procedures and provide written informed consent.

Exclusion criteria were: active tuberculosis or ongoing anti-tuberculosis treatment; acute respiratory infection or exacerbation within four weeks before enrollment; unstable cardiovascular disease or severe neurological or musculoskeletal disorders precluding exercise training; contraindications to LLLT or DO; inability or unwillingness to comply with the rehabilitation program or study assessments; refusal or inability to provide written informed consent.

#### 2.2.2. Baseline Clinical and Demographic Assessment

At baseline, a set of demographic, anthropometric, clinical, and respiratory variables was measured in all participants. The following variables were analyzed: age, gender, body mass index (BMI), pulmonary function tests (PFT), and ventilatory pattern. PFT consisted of the percentage of forced expiratory volume (FEV1), percentage of forced vital capacity (FVC), and FEV1/FVC ratio. The participants were divided into four groups based on the obtained PFT results: normative, obstructive, restrictive, or mixed ventilatory pattern.

The demographic, anthropometric, and clinical characteristics of the population under the experimental and control interventions were compared at baseline to determine whether the two groups were comparable or not. These variables were controlled for in the statistical models of the analysis as potential confounders.

### 2.3. Outcome Measures

Outcome measures were assessed at baseline (T1), before initiation of the rehabilitation program, and after completion of the eight-week intervention (T2), using standardized assessment procedures. Health-related quality of life constituted the primary outcome domain and was assessed using the EUROHIS-QOL-8 and the Physical Health domain of the WHOQOL-BREF. Secondary clinical and functional outcomes included functional exercise capacity (6MWD), functional mobility (TUG), dyspnea (mMRC), and perceived exertion (Borg CR10). NLR and PLR were evaluated as exploratory systemic inflammatory indices. 

#### 2.3.1. Clinical Assessment

The clinical assessment included medical history, demographic characteristics, anthropometric measurements, and pulmonary function testing. Age, sex, and place of residence were obtained from the medical interview. Body weight and height were measured during the baseline clinical assessment, and body mass index (BMI) was calculated as weight in kilograms divided by height in meters squared. The participants’ pulmonary function was determined by spirometry using a Mir SPIROLAB III spirometer (MIR—Medical International Research, Rome, Italy) according to the current ATS/ERS recommendations [36].

#### 2.3.2. Functional Assessment

Functional exercise capacity was assessed by the six-minute walk test (6MWT) and functional mobility was evaluated by the Timed Up and Go (TUG) test [18,37,38]. The 6MWD (six-minute walking distance), established in 6MWT, and TUG were measured by using the BTS G-WALK/BTS G-SENSOR 2 wireless inertial sensor (BTS Bioengineering, Milan, Italy), a wearable inertial gait analysis sensor that contains a tri-axial accelerometer, a tri-axial gyroscope, and a magnetometer. This system includes an inertial sensor composed of a triaxial accelerometer, a magnetic sensor, and a tri-axial gyroscope worn by the subject to allow for objective assessment of functional gait analysis. Further details concerning the specifications and procedures can be found in the BTS G-SENSOR 2 hardware manual (Ver. 1.2.2, Document Number: ERGS2-01271-06, 2016) and the BTS G-WALK^®^ User Manual (Ver. 9.0.0, Document Number: ERGSN-01134-20, 2018).

Although the system allows the assessment of various spatiotemporal gait parameters, only 6MWD and TUG were included as functional outcome measures in the present study. Both tests were performed by the same rehabilitation team at baseline and after the intervention. The 6MWT was performed indoors in a 20-m corridor, marked every 2 m with bright colored tape. At each end of the course, a cone was placed. The patients were tested one at a time and asked to walk as far as possible for 6 min. Standardized verbal encouragement (e.g., “Keep going” and “You’re doing well”) was provided at predefined intervals according to the same protocol for all participants, with identical procedures applied in both groups and at both assessment time points (T1 and T2). The distance walked during the 6 min was recorded.

For the TUG test, the participants were asked to stand up from the chair, walk three meters at a comfortable pace, turn around, walk back to the chair, and sit down. The time was registered in seconds it took the participants to finish the task. The patients also received verbal encouragement. The participants were instructed to walk at their own pace and choose their walking pattern to perform the task of crossing the line at the end of the 3-m course, turn around, and walk back to the chair. The assessor provided the participants with the required help if they needed during the test to ensure the safety of the patients. The test started when the patient left the chair and ended when they returned and sat down in the chair.

#### 2.3.3. Symptoms and Health-Related Quality of Life

Dyspnea was assessed using the modified Medical Research Council (mMRC) dyspnea scale, and perceived exertion during physical exercise was assessed using the Borg CR10 scale [39,40].

Health-related quality of life was assessed using the EUROHIS-QOL-8 and the Physical Health domain of the WHOQOL-BREF [14,41]. The EUROHIS-QOL-8 was used as a brief multidimensional measure of overall quality of life, whereas the WHOQOL-BREF Physical Health domain provided a more focused assessment of physical health aspects relevant to rehabilitation, including pain and discomfort, dependence on medical treatment, energy and fatigue, mobility, sleep and rest, activities of daily living, and work capacity. WHOQOL-BREF Physical Health domain scores were calculated according to WHO scoring guidance and transformed to a 0–100 scale, with higher scores indicating better physical health [15]. Both instruments were administered at baseline and after completion of the eight-week rehabilitation program.

#### 2.3.4. Laboratory Assessment

Peripheral venous blood was drawn after overnight fasting at baseline and after the rehabilitation program. Complete blood count was determined using MINDRAY BC-6800 automated cell counter (Mindray Bio-Medical Electronic Co., Shenzhen, China). In particular peripheral blood neutrophil-to-lymphocyte ratio (NLR) was calculated as absolute neutrophils divided by absolute lymphocytes, while platelet to lymphocyte ratio (PLR) was obtained dividing platelet count by absolute lymphocytes value. Furthermore, both inflammatory indexes were considered as possible biomarkers of increased systemic inflammation in PTLD patients.

### 2.4. Rehabilitation Intervention

The rehabilitation program was carried out by the same multidisciplinary rehabilitation team, according to a standardized treatment protocol. Patients assigned to both interventions followed the same structured but individualized re-habilitation program, developed on the basis of their functional status, ventilatory impairment and exercise capacity. The program consisted of a kinesiotherapy supervision followed by a home-based exercise training. In particular, in the SG, LLLT and DO were associated as adjuvant therapies to the kinesiotherapy.

#### 2.4.1. Structured Kinesiotherapy (Both Groups)

The structured kinesiotherapy protocol was identical for both study groups (Table 1). The supervised rehabilitation phase lasted two weeks and consisted of ten sessions of 40–60 min. This phase was followed by a six-week home-based exercise program supervised through scheduled outpatient follow-up.

Exercise prescription was individualized according to baseline functional status, ventilatory impairment, exercise capacity, symptom response, and exercise tolerance. During aerobic training, progression was achieved by modifying one training variable at a time, such as duration, walking or cycling workload, or interval structure, while maintaining a target Borg CR10 rating of approximately 3–4/10 and stable peripheral oxygen saturation and heart rate. Strength training generally started with 1–2 sets of 8–12 repetitions and progressed to 2–3 sets as tolerated, with resistance increased only when symptoms and oxygenation remained stable. Breathing, thoracic mobility, balance, and gait exercises were adapted according to individual symptom burden and functional performance. The same progression principles and prescribed kinesiotherapy dose were applied in both groups; however, because exercise intensity and progression were individualized, identical prescribed protocols did not necessarily imply identical absolute workloads for every participant. The six-week home-based program was prescribed identically in both groups at approximately 30 min/day, 5–6 days/week. Adherence was monitored through telephone contact every other day and twice-weekly outpatient follow-up. A predefined adherence threshold of ≥80% was required for inclusion in the final analysis. Three participants in the SG and two participants in the CG did not meet this threshold and were therefore not included in the complete-case analysis. All participants included in the final analysis met the ≥80% adherence criterion. However, home-exercise adherence was not recorded as a continuous session-by-session percentage, and therefore, the exact cumulative exercise dose could not be formally compared between groups.

#### 2.4.2. Adjunctive LLLT and DO

Only participants allocated to the SG received adjunctive LLLT and DO. Both modalities were administered immediately before each supervised kinesiotherapy session, based on the rationale that improving thoracic soft-tissue mobility and reducing musculoskeletal discomfort [29,30,31,32,33,34,35] may enhance patients’ ability to perform the prescribed rehabilitation exercises.

LLLT was administered using the ASTAR PhysioGo 500I/501I system (ASTAR Sp. z o.o., Bielsko-Biała, Poland) equipped with an infrared point probe. Treatment parameters included a wavelength of 808 nm, an output power of 100 mW, continuous emission mode, and an energy density of 7 J/cm^2^. The probe was applied in direct contact with the skin, perpendicular to the treatment surface, over three predefined dorsal paravertebral irradiation points on each side, corresponding to the intercostal spaces within the T5–T8 thoracic region. Thus, six irradiation points were treated per session, distributed symmetrically over the right and left paravertebral thoracic regions. Each irradiation point covered an average area of approximately 3 cm^2^ (approximately 1–5 cm^2^), corresponding to an estimated total treated area of approximately 18 cm^2^ per session. The irradiation time was automatically calculated by the device according to the selected treatment parameters. Each treatment session lasted 15 min and was administered once daily during the supervised rehabilitation phase, for a total of 10 treatment sessions. The 808-nm wavelength was selected because near-infrared photobiomodulation provides relatively greater penetration into deeper soft tissues and has been used in previous experimental and clinical studies involving muscle and respiratory-related applications. The energy density of 7 J/cm^2^ was selected within the therapeutic range commonly used for photobiomodulation and in accordance with the predefined device-based clinical protocol [27,28,29,30,31]. Following LLLT, participants in the SG received DO therapy using the DOP1.1 INDIVID device (Physiomed Elektromedizin AG, Schnaittach, Germany) equipped with a 5-cm manual applicator. Treatment was applied bilaterally to the dorsal paravertebral region corresponding to the anatomical projection of the pulmonary hila. The applicator was maintained in direct contact with the skin and moved continuously over the treatment area. Each session consisted of an initial 150-Hz phase for 10 min, followed by a 30-Hz phase for 10 min. The sequential frequency protocol was selected according to the device-based therapeutic approach and the intended treatment objectives. The initial higher-frequency phase (150 Hz) was used for superficial tissue mobilization and facilitation of local circulation, whereas the subsequent lower-frequency phase (30 Hz) was intended to provide slower, deeper oscillatory stimulation aimed at soft-tissue relaxation and mobilization. Treatment was administered daily during the supervised rehabilitation period.

#### 2.4.3. Safety Monitoring

Before each treatment session, each participant was evaluated for acute respiratory tract deterioration, fever, cardiovascular instability, skin lesions, and contraindications to LLLT and deep oscillation. During the therapeutic exercises, peripheral oxygen saturation, heart rate, dyspnea, and exercise performance were also monitored. The appearance of any adverse effects such as dermatological adverse events, discomfort, dizziness, worsening of symptoms and intolerance to treatment were recorded prospectively. No contraindications to the treatment were found and no adverse events or complications were registered.

### 2.5. Statistical Analysis

Statistical analyses were performed using Python 3.13.5 (Python Software Foundation, Wilmington, DE, USA), together with the SciPy 1.17.0 and statsmodels 0.14.6 libraries. The database was screened for missing values, range errors, exact duplicate records, and internal consistency. Recalculated NLR and PLR values, derived from the original hematological measurements, were used for all subsequent analyses.

Continuous variables were summarized as mean ± standard deviation (SD) or median and interquartile range (IQR), according to their distribution and measurement scale, whereas categorical variables were presented as frequencies and percentages. Normality was assessed using the Shapiro–Wilk test and visual inspection of the distributions. Baseline differences between the SG and the CG were evaluated using Welch’s *t*-test, the Mann–Whitney *U* test, Pearson’s chi-square test, or Fisher’s exact test, as appropriate.

Standardized mean differences (SMDs) were calculated to assess baseline balance between groups.

Because the study employed a non-randomized design and baseline imbalances were identified for selected outcome variables, baseline-adjusted analyses were prespecified as the primary between-group analytical approach. For each longitudinal outcome, the mean within-group change from baseline to post-intervention and its corresponding 95% confidence interval (CI) were calculated. The principal between-group effect was estimated using analysis of covariance (ANCOVA), in which the post-intervention value was modeled as the dependent variable, treatment group as the independent variable, and the corresponding baseline value as a covariate. HC3 robust standard errors were applied to account for potential heteroscedasticity, and adjusted between-group estimates were reported together with their 95% CIs.

Model assumptions were evaluated by inspection of residual distributions and residual-versus-fitted plots. Because the mMRC and Borg CR10 scales are ordinal measures, rank-based analyses of the change scores were additionally performed as prespecified sensitivity analyses. Standardized between-group effect sizes were calculated using Hedges’ *g*, based on the difference between the mean change scores in the two groups and the pooled standard deviation of the individual change scores. Hedges’ *g* was selected because it incorporates a correction for small-sample bias. Effect sizes were oriented so that positive values indicated an effect favoring the combined-intervention group. For outcomes in which lower values represented improvement (TUG, mMRC, Borg CR10, NLR, and PLR), the direction of the effect size was reversed accordingly. Absolute values of approximately 0.20, 0.50, and 0.80 were interpreted as small, moderate, and large effects, respectively. Effect-size estimates were reported together with their 95% CIs. Because standardized effect sizes are sensitive to the variability of individual change scores, particularly large Hedges’ g estimates were additionally interpreted alongside the corresponding absolute between-group differences and their 95% confidence intervals.

The primary analysis included 65 participants with complete baseline and post-intervention assessments (SG, *n*= 33; CG, *n*= 32). Eight prespecified longitudinal outcomes were evaluated: 6MWD, TUG, mMRC, Borg CR10, EUROHIS-QOL-8, the WHOQOL-BREF physical-domain score, NLR, and PLR. To control the false discovery rate across these outcomes, the Benjamini–Hochberg procedure was applied. Additional sensitivity analyses adjusted for age, sex, body mass index (BMI), disease duration, and ventilatory pattern. Two-sided *p* values < 0.05 were considered nominally significant, whereas Benjamini–Hochberg-adjusted *q* values < 0.05 were considered statistically significant after correction for multiple testing. The primary analysis therefore constituted a complete-case analysis, because only participants with complete baseline and post-intervention data who met the predefined adherence criterion were included.

Exploratory associations among the eight improvement scores were evaluated separately within each group using two-tailed Spearman rank correlations because several change-score distributions were non-normal and mMRC and Borg CR10 are ordinal scales. All change scores were oriented so that positive values indicated improvement (T2 minus T1 for 6MWD and quality-of-life outcomes; T1 minus T2 for TUG, mMRC, Borg CR10, NLR, and PLR). The Benjamini–Hochberg procedure was applied separately to the 28 pairwise correlations in each group. Correlations with an absolute Spearman coefficient (|*rho*|) of at least 0.50 were considered moderate to strong, while statistical significance was determined using *q* < 0.05.

No formal a priori sample-size calculation was performed. The initial recruitment size was determined by the number of consecutive eligible patients available during the predefined study period rather than by a statistically derived target. After attrition, the primary analysis included 65 participants with complete follow-up. To characterize the approximate magnitude of effect detectable with this available sample, a post hoc minimum detectable effect sensitivity analysis was performed. Assuming a two-sided significance level of 0.05 and 80% statistical power, the available sample corresponded approximately to a standardized between-group effect of Cohen’s *d* = 0.71 (Hedges’ *g* = 0.70) under an independent two-group comparison. This calculation was intended only as a descriptive sensitivity estimate and should not be interpreted as equivalent to an a priori power analysis or as evidence that the study was adequately powered for all secondary or exploratory outcomes.

## 3. Results

### 3.1. Baseline Characteristics

Baseline characteristics of the study population are presented in Table 2. The study and control groups were generally comparable with respect to demographic, anthropometric, clinical, and functional characteristics at baseline. No statistically significant between-group differences were observed for age, sex, place of residence, BMI, disease duration, ventilatory pattern, 6MWD, TUG performance, mMRC dyspnea score, WHOQOL-BREF physical-domain score, NLR, or PLR.

### 3.2. Overall Changes Following Rehabilitation

Within-group changes and baseline-adjusted between-group comparisons are presented in Table 3.

Both groups showed favorable changes following the rehabilitation program, with larger changes observed in the SG. Baseline-adjusted ANCOVA identified statistically significant between-group differences associated with group allocation across all evaluated outcomes, including health-related quality of life, functional exercise capacity, functional mobility, dyspnea, perceived exertion, and the exploratory inflammatory indices (all *q* < 0.001 after Benjamini–Hochberg correction). Although mMRC and Borg CR10 are ordinal scales and are summarized as median [IQR] in the baseline characteristics table, mean ± SD values are presented in Table 3 to correspond with the mean-change, ANCOVA, and standardized effect-size analyses.

Rank-based sensitivity analyses were additionally performed for both outcomes.

### 3.3. Quality-of-Life Outcomes

**Figure 2:**
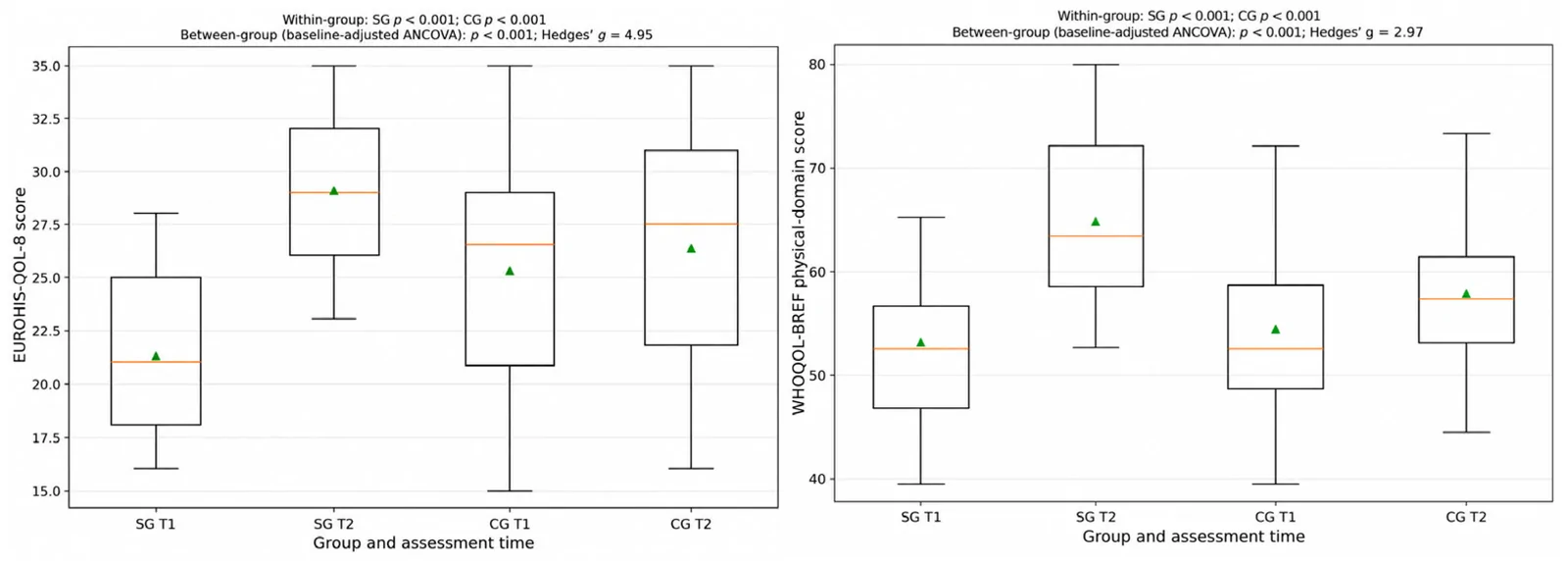
Baseline and post-intervention quality-of-life outcomes in the study and control groups.

Figure 2 presents the results of the analysis of the quality-of-life outcomes after rehabilitation.

Quality-of-life scores improved in both groups, with larger changes observed in the SG. After adjustment for baseline values, group allocation was associated with significant between-group differences in EUROHIS-QOL-8 (adjusted mean difference [AMD]: 6.46; 95% CI: 5.73 to 7.19; *p* < 0.001; *q* < 0.001) and WHOQOL-BREF Physical Health scores (AMD: 8.86; 95% CI: 7.39 to 10.33; *p* < 0.001; *q* < 0.001). Very large standardized between-group effect sizes were observed for both outcomes (Hedges’ *g* = 4.95 and 2.97, respectively). These findings indicate an association between allocation to the combined-intervention group and greater improvement in quality of life, but they do not establish causality. Because Hedges’ *g* standardizes the between-group difference in change by the pooled variability of individual change scores, the unusually large values may partly reflect relatively homogeneous within-group changes and should not be interpreted as proportional causal treatment effects.

### 3.4. Functional and Clinical Outcomes

#### 3.4.1. Functional Outcomes

**Figure 3:**
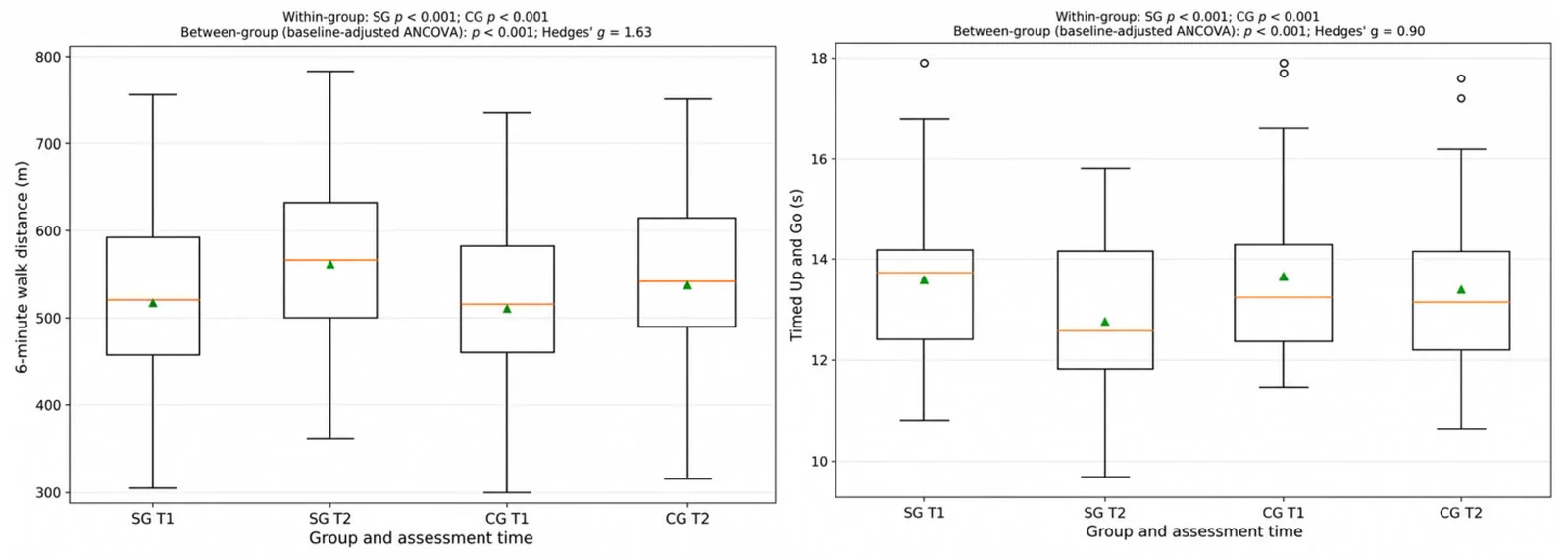
Functional exercise capacity outcomes at baseline and post-intervention.

Functional outcomes are presented in Figure 3. Improvements in functional exercise capacity and mobility were observed in both groups with consistently greater improvements in the SG. Adjusted analysis of covariance revealed that the mean differences between the groups were significant for the 6-min walk test (adjusted mean difference 14.69, 95% CI 10.57 to 18.81; *p* < 0.001; *q* < 0.001) and Timed Up and Go (adjusted mean difference −0.62, 95% CI −0.94 to −0.30; *p* < 0.001; *q* < 0.001). Large standardized effect sizes were found for both outcomes (Hedges’ *g* = 1.63 and 0.90, respectively). For the 6MWD, the mean within-group improvement was 41.91 m in the SG and 27.47 m in the CG, whereas the baseline-adjusted incremental between-group difference was 14.69 m (95% CI 10.57–18.81).

#### 3.4.2. Symptoms

**Figure 4:**
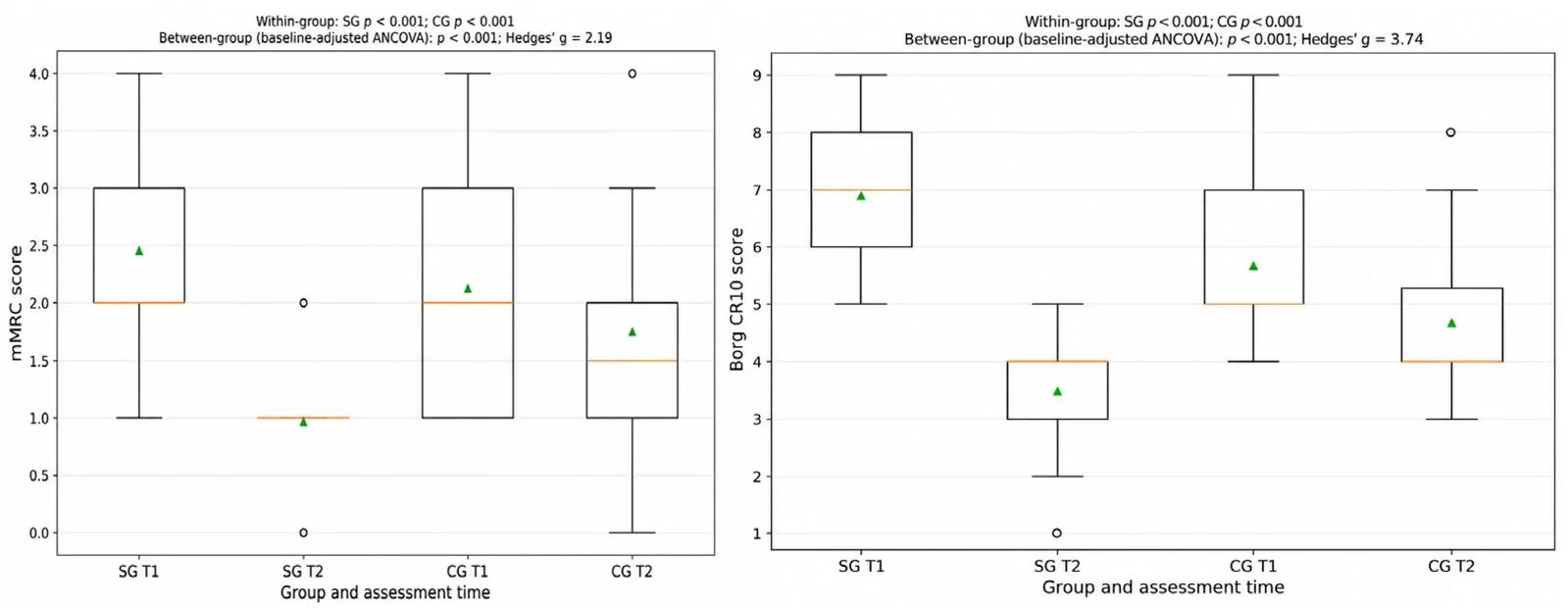
Dyspnea and perceived exertion at baseline and post-intervention.

Changes in dyspnea and perceived exertion are shown in Figure 4. The combined intervention was associated with significantly greater reductions in both dyspnea (mMRC) and perceived exertion (Borg CR10) compared to the structured kinesiotherapy (all *q* < 0.001). Very large standardized effect estimates were observed for both outcomes (Hedges’ *g* = 2.19 for mMRC and 3.74 for Borg CR10); however, these values should be interpreted cautiously because standardized change-score effects are sensitive to the within-group variability of change and do not, by themselves, indicate proportionally large causal treatment effects. The results of the rank-based sensitivity analysis were consistent with those of the primary analysis.

#### 3.4.3. Exploratory Biomarkers

Exploratory inflammatory indices also improved following rehabilitation. After recalculation from the absolute blood-cell counts, both NLR and PLR decreased in the two groups, with significantly greater reductions observed in the SG after baseline adjustment (both *q* < 0.001). Standardized effect sizes were large for both indices (Hedges’ *g* = 1.25 for NLR and 1.09 for PLR). Because these biomarkers were evaluated as exploratory outcomes, the observed changes should not be interpreted as evidence of a modality-specific anti-inflammatory mechanism.

Sensitivity analyses additionally adjusted for age, sex, body mass index, disease duration, and ventilatory pattern produced estimates of the same direction and similar magnitude for all outcomes, confirming the robustness of the primary analyses.

### 3.5. Exploratory Correlation Analysis

**Figure 5:**
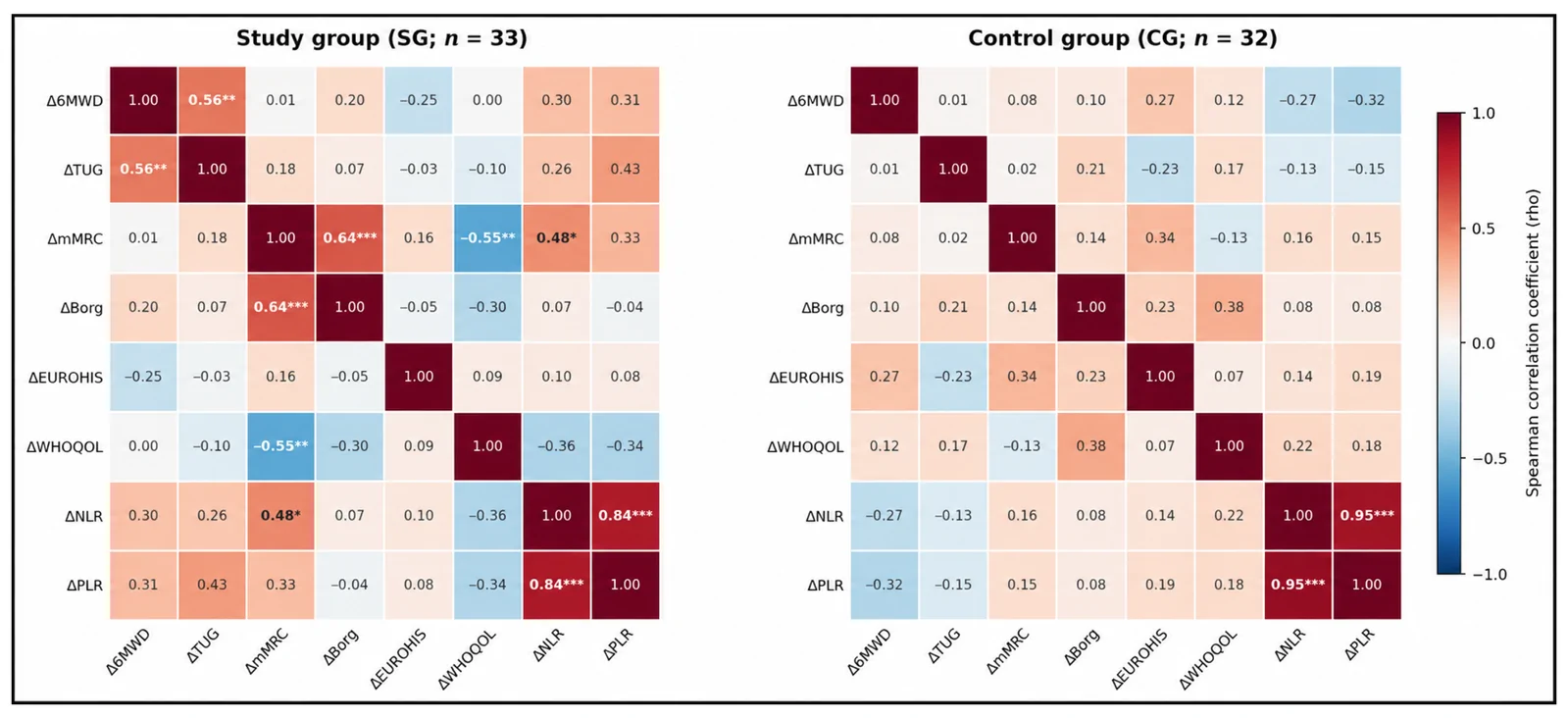
Heatmaps of two-tailed Spearman correlations among clinical improvement scores in the study group (SG) and control group (CG). Red indicates positive associations and blue indicates inverse associations; color intensity reflects correlation magnitude. All change scores were oriented so that positive values indicate clinical improvement. Significance after within-group Benjamini–Hochberg correction: * *q* < 0.05, ** *q* < 0.01, *** *q* < 0.001.

Exploratory correlations between improvements are displayed for the study group and control group separately in Table 4 and Table 5 and also graphically in Figure 5.

In the SG, decreases in NLR and PLR were strongly correlated (*rho* = 0.837, *p* < 0.001, *q* < 0.001). Greater improvement in mMRC was associated with greater improvement in Borg CR10 (*rho* = 0.645, *p* < 0.001, *q* < 0.001), whereas the gain in 6MWD was correlated with improvement in TUG performance (*rho* = 0.556, *p* = 0.001, q = 0.006). In addition, improvement in mMRC was inversely related to the WHOQOL-BREF physical-domain gain (*rho* = −0.550, *p* = 0.001, *q* = 0.006) and positively associated with reduction in NLR (*rho* = 0.485, *p* = 0.004, *q* = 0.024).

In the CG, the only significant correlation after false-discovery-rate adjustment was observed between decreases in NLR and PLR (*rho* = 0.947, *p* < 0.001, *q* < 0.001), whereas all the other associations had *q* ≥ 0.458. These correlation analyses were exploratory and should not be interpreted as evidence of causal or mechanistic relationships between changes in inflammatory indices and clinical or functional outcomes.

## 4. Discussion

This prospective controlled study examined whether receipt of adjunctive LLLT and DO alongside structured pulmonary rehabilitation was associated with greater clinical and functional improvements than structured rehabilitation alone in patients with clinically stable PTLD. The observed between-group differences suggest a potential additional association of the adjunctive modalities with rehabilitation outcomes; however, because allocation was non-randomized, these findings should not be interpreted as evidence of a causal treatment effect. Though photobiomodulation therapy has been widely studied in musculoskeletal and exercise physiology and its mechanisms of action on muscle performance and fatigue have been partially elucidated [42,43], there is a paucity of research on its use in respiratory rehabilitation. A systematic review and meta-analysis suggested that photobiomodulation therapy may have beneficial effects on respiratory muscle performance and exercise-related outcomes in the context of pulmonary rehabilitation, although the evidence remains limited and heterogeneous [31]. A placebo-controlled double-blind crossover trial demonstrated that PBM therapy of respiratory muscles acutely enhances exercise performance capability while having no significant effects on pulmonary function tests, respiratory-muscle strength or thoracoabdominal mobility [30]. Recently, a narrative review concluded that PBM has shown promising effects on dyspnea, exercise tolerance, muscle fatigue, and possibly inflammatory processes in patients with respiratory diseases, but evidence is heterogeneous with regard to the wavelengths, dosimetry, sites of application, treatment schedules, and treatment duration [31]. Because of the variations in treatment protocols, the clinical relevance of the benefits of PBM therapy for patients with PTLD remains unclear.

Further evidence on the mechanisms by which DO improves the outcomes of pulmonary rehabilitation is lacking; preliminary data from musculoskeletal and connective-tissue research suggest that DO may be beneficial in the management of inflammatory pain, soft-tissue mobility restrictions, edema and lymphatic dysfunction [44]. To the best of our knowledge, there are no published studies on the effects of DO on respiratory symptoms or functional capacity in patients with PTLD. In this context, the possible additive benefits of DO and LLLT observed in this study might be mediated through an enhanced tolerance to active pulmonary rehabilitation in patients with PTLD. In patients who report discomfort associated with thoracic expansion, paravertebral muscle tightness, pain and soft-tissue restriction, the analgesic, anti-inflammatory and muscle-relaxant effects of DO and LLLT might facilitate engagement in active rehabilitation, including breathing exercises, thoracic mobility training, walking, strengthening and other exercises [41,44,45]. Accordingly, increased adherence to active rehabilitation might explain the better exercise performance, improved functional mobility, dyspnea scores, perceived exertion and health-related quality of life observed in the combined-intervention group in this study. Evidence from other severe respiratory conditions also supports the importance of structured, exercise-based rehabilitation, with functional benefits reported following comprehensive respiratory rehabilitation after lung transplantation [46].

It should be noted that these mechanisms of action are hypothetical: the concurrent application of PBM therapy and DO precludes the assessment of their independent effects, and the present study did not measure direct effects on respiratory mechanics, chest mobility, thoracoabdominal synchronization, respiratory-muscle activation patterns, local tissue response or pain. Therefore, the current findings are compatible with the possibility that adjunctive electrotherapeutic interventions are associated with greater improvements during an active rehabilitation program; however, the non-randomized design does not demonstrate that these modalities caused the observed differences. In this context, the current findings in patients with PTLD are consistent with the evidence from the broader literature on the benefits of individualized pulmonary rehabilitation in patients with severe respiratory conditions. Individualized training, breathing exercises, and mobilization and strengthening techniques, along with patient education and active self-management, should constitute the core of pulmonary rehabilitation in patients with PTLD. The importance of individualized rehabilitation strategies has also been emphasized in other chronic conditions characterized by persistent functional impairment, where multimodal management and non-pharmacological interventions may contribute to symptom control and functional recovery [47].

In the context of an active rehabilitation program, LLLT and DO might provide additional benefits for patients with persistent symptoms and limitations during physical activity. It should be noted that the evidence for the benefits of these adjunctive methods is insufficient to recommend them as standard of care, and their potential application should consider the volume, equipment, personnel and expertise required, as well as the risks of reduced adherence to the main active interventions. As previously discussed, DO and PBM therapy might help patients with PTLD who have persistent physical limitations during rehabilitation, for instance, due to pain, muscle fatigue or soft-tissue restriction [44]. However, the current evidence is limited to small-scale studies and primarily concerns musculoskeletal targets. For patients with PTLD, evidence of the benefits of physiotherapeutic adjuncts to pulmonary rehabilitation, especially in terms of adherence, is lacking.

The clinical relevance of the observed effects should be considered separately from their statistical significance. The improvement in 6MWD observed in the combined-intervention group exceeded minimal important difference thresholds of approximately 25–30 m reported in COPD populations, whereas the improvement in the control group was also within this range [48,49]. However, the incremental benefit associated with the combined adjunctive strategy was smaller than these commonly reported thresholds, suggesting that the additional improvement in exercise capacity should be interpreted cautiously despite its statistical significance. For mMRC, a minimal important difference of approximately 0.5–0.6 points has been reported in patients with COPD [50]. The improvement in dyspnea observed in the present study was compatible with this benchmark. However, neither the 6MWD nor the mMRC thresholds cited above have been specifically established in patients with PTLD and should therefore be considered contextual rather than disease-specific benchmarks.

The very large standardized effect estimates observed for EUROHIS-QOL-8, Borg CR10, WHOQOL-BREF Physical Health, and mMRC require additional caution. Hedges’ g was calculated from the between-group difference in mean change scores divided by the pooled standard deviation of the individual change scores; consequently, relatively homogeneous within-group changes produce a small denominator and can yield very large standardized estimates. In addition, the non-randomized allocation, the baseline imbalances for Borg CR10 and EUROHIS-QOL-8, and the absence of sham procedures may have increased the observed between-group contrasts through selection effects, regression to the mean, treatment expectations, and additional therapist contact. The relatively small sample may also make standardized estimates less stable. Therefore, these unusually large Hedges’ g values should not be interpreted as evidence of equivalently large causal treatment effects, but rather together with the absolute between-group differences, 95% confidence intervals, and clinically meaningful thresholds where available.

Beyond functional performance, this study also explored the potential benefits of additional physiotherapeutic interventions on inflammatory processes in patients with PTLD. Both study groups showed reductions in two exploratory inflammatory markers, the NLR and PLR, with more pronounced decreases in the combined-intervention group. These findings should be considered tentative, as there is limited published information concerning the role of inflammation in PTLD. However, the concept of persistent low-grade systemic inflammation as a mechanism by which PTLD contributes to reduced physical performance and postulated links between chronic inflammation and impaired quality of life are increasingly attracting scientific and clinical attention [12]. Evidence from other clinical populations also suggests a potential relationship between exercise-based rehabilitation, functional improvement, and systemic inflammatory status. In overweight and obese children, a 12-week individualized kinesiotherapy program was associated with improvements in functional performance and reductions in inflammatory markers, including C-reactive protein and leptin [51]. Experimental data indicate that photobiomodulation may decrease neutrophil infiltration and production of pro-inflammatory cytokines such as IL-1β, IL-6 and TNF-α while promoting anti-inflammatory responses, including increased IL-10 production, in models of COPD, acute lung injury and eosinophilic allergic airway inflammation [52,53,54]. The effects of DO on systemic inflammation have not been studied, but some evidence suggests that DO can improve lymphatic drainage and local circulation [46]. The greater reductions in NLR and PLR observed in the combined-intervention group should be interpreted as exploratory findings. Although these indices are commonly used as indirect markers of systemic inflammatory status, the present study did not assess cytokines, pulmonary inflammatory activity, respiratory mechanics, or tissue-level responses. Therefore, the observed changes cannot establish an anti-inflammatory mechanism attributable to LLLT, DO, or their combination. The associations between changes in these inflammatory indices and clinical outcomes should likewise be regarded as exploratory and hypothesis-generating rather than as evidence of causal or mechanistic relationships.

However, several notes of caution are needed. First, the current findings concern inflammatory biomarkers that were merely exploratory outcomes in this study. Second, NLR and PLR are indirect measures of systemic inflammation that reflect changes in neutrophil, platelet and lymphocyte counts. These parameters can be affected by a wide array of factors, including concurrent infections, medication use, comorbidities, nutrition, fluid balance and other processes not evaluated in this study. Finally, while experimental data suggest that photobiomodulation might reduce cytokine production and promote anti-inflammatory responses, these data come from animal models of respiratory diseases and cannot be directly extrapolated to patients with PTLD. Therefore, the current findings should be considered tentative and potentially informative only for future intervention trials with larger sample sizes, appropriate control groups and additional inflammatory biomarkers.

In context, the findings of this study add to the growing body of evidence concerning the benefits of pulmonary rehabilitation for patients with PTLD. Despite the persistence of structural lung damage following microbiological cure for tuberculosis, pulmonary rehabilitation has been shown to improve exercise capacity, reduce dyspnea and enhance health-related quality of life in patients with PTLD [12,13,14,22,23]. Similarly, Mtei et al. [41] demonstrated that an 24-week community-based pulmonary rehabilitation program can improve walking performance and respiratory-specific quality of life in patients with PTLD, though conventional lung-function tests showed little change. The present study extends previous work by comparing outcomes in patients receiving structured kinesiotherapy with or without adjunctive LLLT and DO. The observed between-group differences were associated with the combined adjunctive strategy, including differences in functional outcomes and exploratory inflammatory indices; however, these findings should be regarded as hypothesis-generating rather than as evidence of a causal treatment effect.

The study has several strengths. First, the active controlled design allowed direct comparison of outcomes between structured kinesiotherapy alone and structured kinesiotherapy combined with adjunctive LLLT and DO. Second, the study measured a wide range of functional and symptom-related outcomes along with exploratory inflammatory biomarkers using standardized, reliable procedures. Finally, the statistical analysis applied baseline-adjusted analyses, including HC3 robust standard errors, confidence intervals, False Discovery Rate correction and sensitivity analyses, to ensure the validity of the findings. Nevertheless, the study also has limitations, the most significant of which concern the lack of randomization and resultant potential for selection bias. While the study adjusted for baseline differences between participants, baseline characteristics of the two groups differed for Borg CR10 and EUROHIS-QOL-8 scores. In particular, the higher baseline Borg CR10 scores and lower EUROHIS-QOL-8 scores in the study group may have provided greater scope for improvement and may have contributed to regression-to-the-mean effects. Although baseline-adjusted ANCOVA reduced the influence of these measured differences, residual confounding cannot be excluded because treatment allocation was not randomized. Additionally, the study used an open-label design, and there was no sham intervention for the control group; therefore, the effects of attention and expectations cannot be entirely excluded, particularly for self-reported outcomes. Another limitation concerns the interpretation of clinical relevance. The MCID thresholds used to contextualize the 6MWD and mMRC findings were derived primarily from other chronic respiratory disease populations and have not been specifically validated in PTLD. In addition, PTLD-specific MCID thresholds have not been established for several outcomes assessed in this study, including TUG, Borg CR10, EUROHIS-QOL-8, and the WHOQOL-BREF Physical Health domain. Statistical significance and standardized effect sizes should therefore not be considered equivalent to clinically important benefit.

The concurrent administration of LLLT and DO represents an additional limitation, as the study evaluated the two modalities as a combined adjunctive strategy and cannot determine their independent contributions or potential additive or synergistic interactions. Distinguishing these effects would require an appropriately designed multi-arm or factorial randomized controlled trial.

The non-randomized allocation procedure also warrants consideration. Because group allocation depended on treatment availability and participation feasibility, systematic differences between groups may have persisted despite adjustment for measured baseline characteristics, and residual confounding from unmeasured factors cannot be excluded. The unusually large standardized effect estimates should likewise be interpreted cautiously, as their magnitude may have been influenced by low change-score variability, baseline imbalances, non-randomized allocation, and non-specific effects associated with the open-label design.

Finally, the primary analysis was restricted to participants with complete baseline and post-intervention assessments who attended at least 80% of the rehabilitation program, introducing the possibility of attrition bias. Completers may have differed systematically from non-completers in motivation, adherence, functional status, or ability to attend supervised sessions. Selective retention may therefore have influenced the magnitude of the observed between-group differences.

Although oxygen saturation was monitored during supervised exercise sessions for safety purposes, it was not prespecified as an efficacy outcome and was therefore not used to assess treatment response. The study primarily evaluated functional and patient-reported outcomes and did not include serial assessment of gas exchange or other objective physiological measures specifically designed to quantify changes in respiratory function. Consequently, the observed improvements in exercise capacity, symptoms, and health-related quality of life should not be interpreted as evidence of improvement in pulmonary physiology. Even if the same therapeutic classes for PTLD were used in both groups and participants’ prescribed treatments remained unchanged during the study period, pharmacological management was individualized and concomitant medication regimens were not formally incorporated into the comparative analyses. Therefore, residual confounding related to individual differences in background pharmacological treatment cannot be completely excluded and should be considered when interpreting the observed between-group differences. The study did not include sham LLLT or DO procedures, and participants and therapists were not blinded to treatment allocation. Consequently, the study group received additional treatment-related contact and procedures that were not matched in the control group. Expectation effects, increased therapist interaction, and perceived treatment novelty may therefore have contributed to the observed between-group differences, particularly for patient-reported outcomes such as health-related quality of life, dyspnea, and perceived exertion. A sham-controlled design would be required to better distinguish these non-specific effects from the specific effects of the adjunctive intervention. Further limitations concern the lack of a priori power analysis, the use of an exploratory approach for secondary outcomes and the relatively small sample size. Finally, the study was conducted at a single center and did not include long-term follow-up. Disease-specific respiratory quality-of-life questionnaires, objective measurements of respiratory mechanics and chest-wall kinematics, instrumented gait-analysis, and long-term pulmonary-function assessments were not performed. Because no a priori sample-size calculation was performed and the available cohort was determined by consecutive recruitment during the study period, the study may have been underpowered to detect smaller effects for some secondary and exploratory outcomes. The post hoc sensitivity calculation should therefore not be interpreted as demonstrating adequate statistical power for these outcomes. Although the prescribed kinesiotherapy protocol and home-exercise program were identical in both groups and all participants included in the final analysis met the predefined ≥80% adherence threshold, adherence to the six-week home-based program was not quantified as a continuous session-by-session measure. Therefore, differences in the actual cumulative exercise dose between groups cannot be completely excluded and may have contributed to the observed between-group differences. The study used inflammatory biomarkers (NLR and PLR) as surrogate endpoints, which should be validated in future studies.

Therefore, the applicability of the findings to more general populations of patients with PTLD is limited, and future trials are needed to confirm the findings in larger, multicenter studies. The within-group correlations should also be considered exploratory, as they concern relatively small samples and multiple comparisons. Thus, in future research, larger cohort studies and clinical trials with sufficient statistical power should be conducted to determine whether the benefits of pulmonary rehabilitation in patients with PTLD are modified by additional physiotherapeutic interventions or can be enhanced by increasing the duration of the individual rehabilitation program. Further research should also elucidate the mechanisms by which adjunctive physiotherapeutic interventions affect functional performance and inflammatory biomarkers. It should be noted that the strong correlation between changes in NLR and PLR may be partially explained by the fact that these two markers share lymphocytes as a common denominator.

Finally, future randomized controlled trials should specify a primary outcome and examine the differential benefits of LLLT and DO using sham or contact-matched procedures. Longer follow-up, comprehensive safety assessment, disease-specific quality-of-life assessments, objective evaluation of respiratory mechanics and broader inflammatory panels will also be necessary to determine whether the benefits of these interventions observed in the present trial can be reproduced in future studies.

## 5. Conclusions

Structured pulmonary rehabilitation was associated with improvements in functional capacity, mobility, dyspnea, perceived exertion, and health-related quality of life in patients with clinically stable post-tuberculosis lung disease. Patients allocated to structured kinesiotherapy combined with LLLT and DO showed greater improvements than those receiving structured kinesiotherapy alone; however, because allocation was non-randomized, these between-group differences should be interpreted as associative and not as evidence of a causal effect of the adjunctive modalities. Reductions in NLR and PLR were exploratory and should not be interpreted as evidence of a specific anti-inflammatory mechanism. Future randomized controlled trials are needed to further elucidate the independent role of the interventions used, determine their relevance to the management of PTLD, and establish their place in pulmonary rehabilitation programs.

## Figures and Tables

**Table 1:** Components of the structured kinesiotherapy program and progression criteria applied during supervised rehabilitation.

Component	Supervised Prescription	Progression/Safety Criteria
Breathing and airway- clearance exercises	Breathing control, diaphragmatic breathing, thoracic expansion, pursed-lip breathing, thoracic mobility; huff/cough techniques when secretions were present.	Adapted to symptom burden; pauses and anti- dyspnea positions used when required.
Aerobic training	Walking or cycle-based interval/continuous training, about 20–40 min including warm-up and cool-down.	Progress only one variable per session; target Borg 3–4/10; monitor SpO_2_ and heart rate.
Strength training	Upper- and lower-limb exercises, initially 1–2 sets of 8–12 repetitions, progressing to 2–3 sets as tolerated.	Exhale during effort; increase resistance only with stable symptoms and oxygenation.
Balance and gait training	Weight shifts, directional changes, obstacle negotiation, tandem walking, step-ups and supported single-leg stance.	Close guarding and external support as needed.
Education and self- management	Pacing, energy conservation, hydration, airway hygiene, recognition of warning signs and home-exercise progression.	Brief education integrated into each supervised session.
Home program	30 min/day, 5–6 days/week for 6 weeks: breathing, strength, balance and aerobic walking.	Telephone (every other day) and twice weekly outpatient

**Table 2:** Baseline characteristics of the study population.

Variable	SG (*n* = 33)	CG (*n* = 32)	Between-Group *p*	Standardized Difference
Age, years	48.9 ± 14.2	49.0 ± 12.4	0.986	−0.00
Male sex, *n* (%)	20 (60.6)	16 (50.0)	0.459	0.21
Urban residence, *n* (%)	18 (54.5)	16 (50.0)	0.806	0.09
BMI, kg/m^2^	23.85 ± 2.86	23.98 ± 2.84	0.852	−0.05
Disease duration, months	12.0 [6.0–24.0]	12.0 [6.0–23.2]	0.920	−0.09
Ventilatory pattern O/R/M, *n*	1/22/10	1/19/12	0.824	V = 0.08
6MWD, m	517.8 ± 103.1	509.6 ± 101.2	0.747	0.08
TUG, s	13.60 ± 1.50	13.65 ± 1.73	0.893	−0.03
mMRC score	2.0 [2.0–3.0]	2.0 [1.0–3.0]	0.105	0.36
Borg CR10	7.0 [6.0–8.0]	5.0 [5.0–7.0]	<0.001	0.96
EUROHIS-QOL-8	21.3 ± 3.8	25.3 ± 5.6	0.001	−0.84
WHOQOL-BREF physical domain	52.0 ± 7.1	54.0 ± 7.8	0.294	−0.26
NLR	4.12 [3.50–5.00]	4.00 [3.23–4.17]	0.244	0.23
PLR	194.12 [161.11–218.75]	182.35 [147.37–213.33]	0.477	0.17

Data are presented as mean ± SD, median [IQR], or *n* (%), as appropriate. Standardized mean differences (SMDs) are reported for continuous variables, whereas Cramer’s V is reported for categorical variables. NLR and PLR were recalculated from the absolute blood-cell counts. Abbreviations: SG, study group; CG, control group; *n*, number of participants; SD, standard deviation; IQR, interquartile range; SMD, standardized mean difference; BMI, body mass index; O/R/M, obstructive/restrictive/mixed ventilatory pattern; 6MWD, six-minute walk distance; TUG, Timed Up and Go; mMRC, modified Medical Research Council; CR10, Category-Ratio 10 scale; NLR, neutrophil-to-lymphocyte ratio; PLR, platelet-to-lymphocyte ratio.However, participants in the study group reported higher baseline perceived exertion, reflected by higher Borg CR10 scores (median 7.0 [IQR 6.0–8.0] vs. 5.0 [5.0–7.0]; *p* < 0.001; SMD = 0.96), and lower overall health-related quality of life, reflected by lower EUROHIS-QOL-8 scores (21.3 ± 3.8 vs. 25.3 ± 5.6; *p* = 0.001; SMD = −0.84). These baseline imbalances confirmed the appropriateness of the prespecified baseline-adjusted analyses.

**Table 3:** Changes from baseline to post-intervention and baseline-adjusted between-group comparisons.

Outcome	SG T1	SG T2	SG Δ (95% CI)	CG T1	CG T2	CG Δ (95% CI)	Baseline- Adjusted Between-Group Effect (95% CI)	Hedges’ *g* (95% CI)	*p*/*q*
EUROHIS-QOL-8	21.3 ± 3.8	29.1 ± 3.5	7.79 (7.28 to 8.30)	25.3 ± 5.6	26.4 ± 5.6	1.06 (0.61 to 1.51)	6.46 (5.73 to 7.19)	4.95 (3.96 to 5.95)	<0.001/<0.001
WHOQOL-BREF physical domain	52.0 ± 7.1	64.3 ± 8.6	12.27 (11.00 to 13.54)	54.0 ± 7.8	57.3 ± 7.2	3.38 (2.60 to 4.15)	8.86 (7.39 to 10.33)	2.97 (2.26 to 3.68)	<0.001/<0.001
6MWD (m)	517.8 ± 103.1	559.7 ± 98.5	41.91 (39.26 to 44.56)	509.6 ± 101.2	537.0 ± 100.3	27.47 (23.91 to 31.03)	14.69 (10.57 to 18.81)	1.63 (1.07 to 2.20)	<0.001/<0.001
TUG (s)	13.60 ± 1.50	12.73 ± 1.48	−0.87 (−1.19 to −0.54)	13.65 ± 1.73	13.40 ± 1.69	−0.26 (−0.33 to −0.18)	−0.62 (−0.94 to −0.30)	0.90 (0.39 to 1.42)	<0.001/<0.001
mMRC	2.5 ± 0.8	1.0 ± 0.6	−1.48 (−1.66 to −1.30)	2.1 ± 1.0	1.8 ± 1.0	−0.38 (−0.55 to −0.20)	−1.04 (−1.29 to −0.79)	2.19 (1.57 to 2.81)	<0.001/<0.001
Borg CR10	6.9 ± 1.2	3.5 ± 1.1	−3.45 (−3.69 to −3.22)	5.7 ± 1.4	4.7 ± 1.3	−0.97 (−1.20 to −0.74)	−2.21 (−2.63 to −1.79)	3.74 (2.93 to 4.56)	<0.001/<0.001
NLR	4.10 ± 1.11	2.54 ± 0.55	−1.56 (−1.80 to −1.32)	3.84 ± 1.09	3.10 ± 1.13	−0.74 (−0.96 to −0.52)	−0.73 (−1.01 to −0.44)	1.25 (0.71 to 1.78)	<0.001/<0.001
PLR	189.49 ± 42.34	145.71 ± 23.16	−43.78 (−51.61 to −35.95)	182.24 ± 43.30	160.81 ± 42.23	−21.43 (−27.93 to −14.94)	−20.19 (−28.51 to −11.87)	1.09 (0.57 to 1.62)	<0.001/<0.001

T1 and T2 values are mean ± SD; Δ denotes the mean T2–T1 change. Baseline-adjusted effects were estimated by ANCOVA with the post-intervention value as the dependent variable, treatment group as the main predictor, and the corresponding baseline value as a covariate; HC3 robust standard errors were used. Negative adjusted effects favor the SG for TUG, mMRC, Borg CR10, NLR, and PLR. Hedges’ *g* values were oriented so that positive values indicate an effect in favor of the SG. *p* values are nominal ANCOVA values; *q* values were adjusted using the Benjamini–Hochberg procedure. Abbreviations: SG, study group; CG, control group; T1, baseline assessment; T2, post-intervention assessment; CI, confidence interval; SD, standard deviation; 6MWD, six-minute walk distance; TUG, Timed Up and Go; mMRC, modified Medical Research Council; CR10, Category-Ratio 10 scale; NLR, neutrophil-to-lymphocyte ratio; PLR, platelet-to-lymphocyte ratio; ANCOVA, analysis of covariance.

**Table 4:** Spearman correlation matrix for improvement scores in the study group (SG; *n* = 33).

Improvement Score	Δ6MWD	ΔTUG	ΔmMRC	ΔBorg	ΔEUROHIS	ΔWHOQOL	ΔNLR	ΔPLR
Δ6MWD	1.000	0.556 ** <0.001/0.006	0.010 0.958/0.991	0.198 0.269/0.501	−0.250 0.160/0.320	0.002 0.991/0.991	0.301 0.089/0.208	0.308 0.081/0.208
ΔTUG		1.000	0.182 0.311/0.545	0.071 0.696/0.876	−0.027 0.881/0.949	−0.103 0.569/0.838	0.260 0.144/0.310	0.433 0.012/0.056
ΔmMRC			1.000	0.645 *** <0.001/<0.001	0.164 0.360/0.594	−0.550 ** <0.001/0.006	0.485 * 0.004/0.024	0.329 0.062/0.192
ΔBorg				1.000	−0.047 0.797/0.913	−0.301 0.089/0.208	0.065 0.719/0.876	−0.042 0.815/0.913
ΔEUROHIS					1.000	0.091 0.616/0.862	0.103 0.568/0.838	0.076 0.673/0.876
ΔWHO QOL						1.000	−0.357 0.041/0.166	−0.339 0.054/0.187
ΔNLR							1.000	0.837 *** <0.001/<0.001
ΔPLR								1.000

Note: Cells show Spearman *rho* on the first line and nominal *p*/Benjamini–Hochberg-adjusted *q* on the second line. Positive changes denote improvement for every variable. * *q* < 0.05; ** *q* < 0.01; *** *q* < 0.001. Δ6MWD and quality-of-life scores = T2 − T1; ΔTUG, ΔmMRC, ΔBorg, ΔNLR, and ΔPLR = T1 − T2.

**Table 5:** Spearman correlation matrix for improvement scores in the control group (CG; *n* = 32).

Improvement Score	Δ6MWD	ΔTUG	ΔmMRC	ΔBorg	ΔEUROHIS	ΔWHOQOL	ΔNLR	ΔPLR
Δ6MWD	1.000	0.014 0.939/0.939	0.081 0.661/0.763	0.098 0.594/0.756	0.266 0.141/0.661	0.125 0.496/0.661	−0.265 0.142/0.661	−0.321 0.073/0.512
ΔTUG		1.000	0.024 0.894/0.927	0.205 0.259/0.661	−0.228 0.210/0.661	0.170 0.352/0.661	−0.134 0.463/0.661	−0.153 0.404/0.661
ΔmMRC			1.000	0.139 0.448/0.661	0.343 0.054/0.508	−0.128 0.486/0.661	0.165 0.368/0.661	0.151 0.411/0.661
ΔBorg				1.000	0.225 0.215/0.661	0.378 0.033/0.458	0.076 0.681/0.763	0.080 0.664/0.763
ΔEUROHIS					1.000	0.066 0.722/0.777	0.136 0.458/0.661	0.186 0.308/0.661
ΔWHO QOL						1.000	0.217 0.233/0.661	0.185 0.311/0.661
ΔNLR							1.000	0.947 *** <0.001/<0.001
ΔPLR								1.000

Note: Cells show Spearman *rho* on the first line and nominal *p*/Benjamini-Hochberg-adjusted *q* on the second line. Positive changes denote improvement for every variable. *** *q* < 0.001. Δ6MWD and quality-of-life scores = T2 − T1; ΔTUG, ΔmMRC, ΔBorg, ΔNLR, and ΔPLR = T1 − T2.

## Data Availability

The data presented in this study are available from the corresponding author upon reasonable request. The data are not publicly available due to privacy and ethical restrictions related to the protection of participants’ personal information.
